# AI‐organoid integrated systems for biomedical studies and applications

**DOI:** 10.1002/btm2.10641

**Published:** 2024-01-20

**Authors:** Sudhiksha Maramraju, Andrew Kowalczewski, Anirudh Kaza, Xiyuan Liu, Jathin Pranav Singaraju, Mark V. Albert, Zhen Ma, Huaxiao Yang

**Affiliations:** ^1^ Department of Biomedical Engineering University of North Texas Denton Texas USA; ^2^ Texas Academy of Mathematics and Science University of North Texas Denton Texas USA; ^3^ Department of Biomedical & Chemical Engineering Syracuse University Syracuse New York USA; ^4^ BioInspired Institute for Material and Living Systems Syracuse University Syracuse New York USA; ^5^ Department of Mechanical & Aerospace Engineering Syracuse University Syracuse New York USA; ^6^ Department of Computer Science and Engineering University of North Texas Denton Texas USA

**Keywords:** artificial intelligence, deep learning, disease modeling, drug evaluation, human pluripotent stem cells (hPSCs), machine learning, organoid, regenerative medicine

## Abstract

In this review, we explore the growing role of artificial intelligence (AI) in advancing the biomedical applications of human pluripotent stem cell (hPSC)‐derived organoids. Stem cell‐derived organoids, these miniature organ replicas, have become essential tools for disease modeling, drug discovery, and regenerative medicine. However, analyzing the vast and intricate datasets generated from these organoids can be inefficient and error‐prone. AI techniques offer a promising solution to efficiently extract insights and make predictions from diverse data types generated from microscopy images, transcriptomics, metabolomics, and proteomics. This review offers a brief overview of organoid characterization and fundamental concepts in AI while focusing on a comprehensive exploration of AI applications in organoid‐based disease modeling and drug evaluation. It provides insights into the future possibilities of AI in enhancing the quality control of organoid fabrication, label‐free organoid recognition, and three‐dimensional image reconstruction of complex organoid structures. This review presents the challenges and potential solutions in AI‐organoid integration, focusing on the establishment of reliable AI model decision‐making processes and the standardization of organoid research.

**Translational Impact Statement**

The hPSC‐derived organoids have been widely applied in disease modeling, drug evaluation, and regenerative medicine. The differentiation, structure, and function of organoids are characterized by microscopic imaging, transcriptomics, metabolomics, and proteomics by generating large and complex datasets. Currently, most post‐experimental data analysis is inefficient and prone to being human‐biased. To address this issue, AI is found to be an effective approach to learning from substantial amounts of complex data and subsequently deriving the biomedical meaning and applications of hPSC‐derived organoids. Here, we comprehensively reviewed AI‐assisted biomedical and translational research in hPSC‐derived organoids.

## 1 INTRODUCTION

Organoids are three‐dimensional (3D) cell constructs that can be directly derived from human pluripotent stem cells (hPSCs). An organoid contains a complex multicellular cluster of organ‐specific cells that closely resemble the structure and function of an organ.^1^, ^2^ Due to their striking similarity to organs, organoids have been proven to be beneficial for the study of organ development and human diseases.^3^ Researchers are actively developing various organoids by creating an optimal environment with developmental‐relevant biochemical and biophysical cues for stem cells to differentiate and resemble engineered tissues or organs with multiple cell types and intercellular crosstalk, thus increasing the predictive validity related to organ and tissue pathophysiology and function.^4^ The hPSC‐derived organoids have been widely used in drug evaluation, regenerative medicine, and disease modeling in the past decade. The physiological replication of human organs by the hPSC‐derived organoids allows for testing drug responses and efficacies more ethically by replacing animal models in preclinical studies in drug discovery and development.^5^ In regenerative medicine, organoids can also be used to develop new methods to regrow, repair, or replace damaged or diseased tissues.^6^ Moreover, organoid‐based disease models have been applied to study genetic disorders using genetically engineered hPSCs.

In general, hPSC‐organoids are extensively characterized using a broad range of experimental approaches, including transcriptomics, metabolomics, proteomics, single‐cell analysis, and microscopic imaging, which generate large and multimodal datasets that require further analysis and summarization.^2^, ^7^ However, current data analysis methods are highly inefficient because they require researchers to handle large volumes of data with varying levels of complexity manually or semi‐manually. More importantly, as the volume and complexity of the data grow, the conclusions concerning the data become more difficult to obtain,^8^ and human bias and intuition can lead to incorrect or contradictory conclusions in biomedical research and discovery. To address these challenges, several automatic approaches using artificial intelligence (AI) have been enabled due to recent advancements in computer and data science and engineering. AI is capable of augmenting, and in limited cases replacing, human intelligence, improving efficiency, and increasing accuracy with less human bias and subjectivity^9^. Computer vision‐based AI can examine and analyze data from the organoids and be more systematically evaluated than human judgment, resulting in more accurate results that can be utilized in future (pre)clinical trials, diagnoses, and treatments.^10^

This review provides a brief overview and introduction for biomedical researchers with limited AI background on the recent progress in AI‐assisted research of hPSC‐derived organoids. We highlight how the integration of AI and related technologies can accelerate the biomedical research of hPSC‐derived organoids. We begin by introducing the typical methods for characterizing hPSC‐derived organoids, then further introducing the basic knowledge of AI in biomedical studies, and then how AI‐assisted analysis of the complex and large datasets generated from organoid‐specific characterizations further enhances the hPSC‐derived organoid systems. Finally, we discuss the future directions and limitations of AI‐hPSC‐derived organoids integrated system.

## 2 DATA COLLECTION FROM ORGANOID CHARACTERIZATION

**FIGURE 1:**
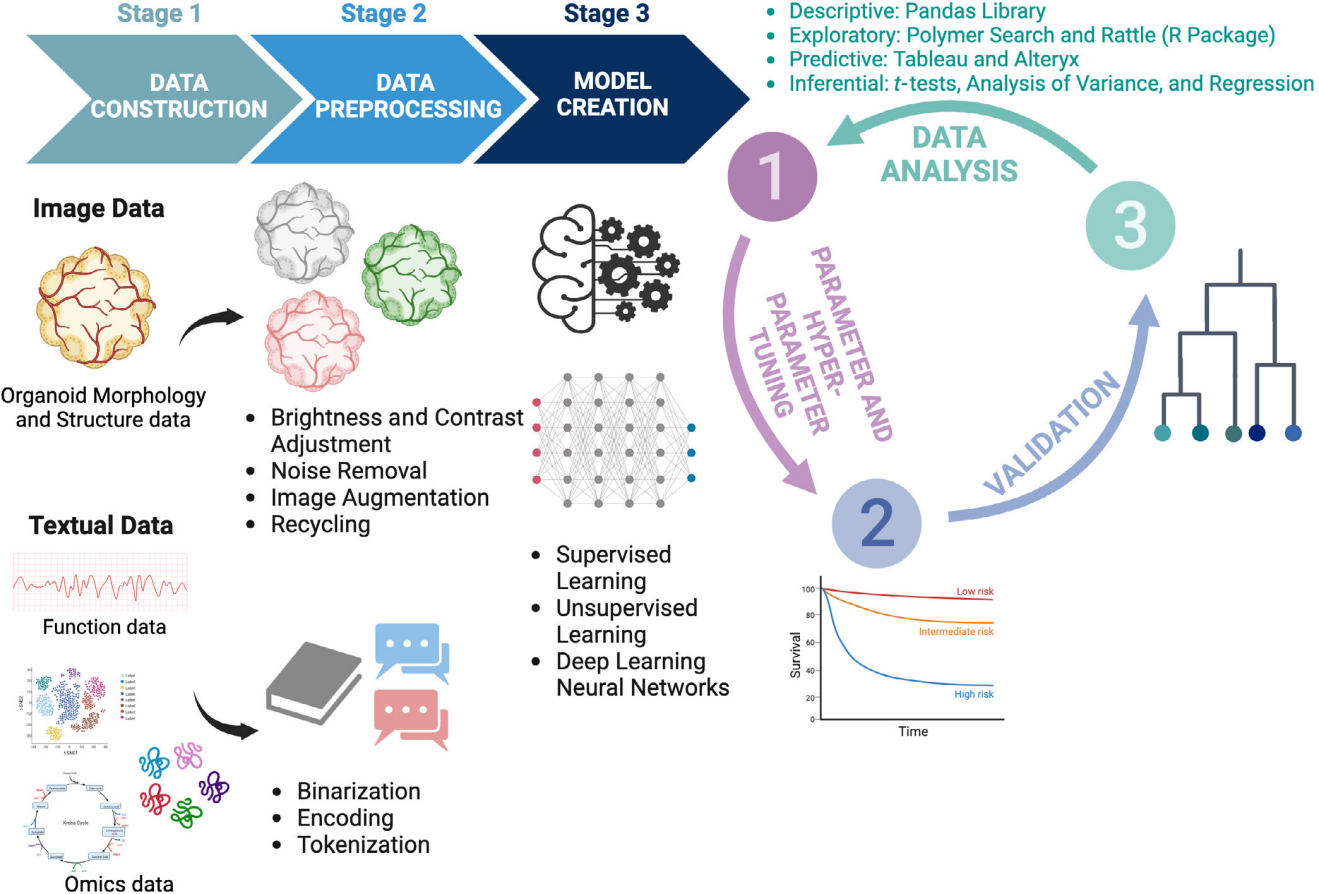
Integration of AI‐organoid system. Step 1: dataset construction from organoid imaging, function measurement, and multiomics; Step 2: data preprocessing based on data type; and Step 3: machine/deep learning model creation with a closed‐loop optimization by parameter/hyperparameter tuning, validation, and data visualization.

The hPSC‐derived organoids are typically characterized by multiomics of transcriptomics, metabolomics, proteomics, and microscopic imaging for organoid functions and morphology/structure (Figure 1). Different types of datasets (e.g., text and image) generated by various characterization methods are further analyzed by AI.

### 2.1 Multiomics analysis

The use of next‐generation RNA sequencing (RNA‐seq) technologies allows researchers to profile transcriptomes^11^ and analyze the resulting datasets to decipher the transcriptional activity of both coding and non‐coding RNAs and target key genes and transcripts.^12^ RNA‐seq analysis is particularly valuable in comparing the differential transcriptome of hPSC‐derived organoids under various conditions, such as different developmental stages, pathological conditions, and treatments at a molecular level.^13^, ^14^ For example, time‐course bulk RNA‐seq has been applied to examine the retinal organoid differentiation from hPSCs, to elucidate the temporal expression of retinal differentiation markers and mRNA alternative splicing occurring during in vivo retinogenesis.^15^ Meanwhile, scRNA‐seq shows great potential in delineating the heterogeneity and specificity of multicellular organoids composed of tens of thousands of individual cells. scRNA‐seq has been used to determine organoid‐to‐organoid variability.^16^ This technology also allows researchers to uncover disease mechanisms that are related to multiple rare cell populations, which are not visible when investigating a large group of cells together.^17^ scRNA‐seq is also used to quantify organoid cell resemblance to primary tissue equivalents in the brain, gut, liver, heart, and kidney and to detect cell‐specific responses to environmental factors and disease situations.^18^, ^19^, ^20^ Additionally, cellular and molecular heterogeneity in brain organoids has been dissected using single‐cell transcriptomics or epigenomics to reveal the complex organization of brain organoids.^21^ Overall, transcriptome profiling and analysis help researchers discover genes that are differentially expressed under diverse contexts, leading to a better understanding of the genes and processes that are linked to developmental and pathophysiological conditions.

Proteomics is the study of these proteins to determine their identity, abundance, and function associated with cells, tissues, or organisms.^22^ Meanwhile, metabolomics analyzes the metabolites are products and intermediates of cellular metabolism that play essential roles in energy conversion, signaling, epigenetic influence, and cofactor activity.^23^ For example, mass spectrometry‐based proteomics was used to study hPSC‐derived small intestine organoids to distinguish between crypt‐like and villus‐like formations. This approach successfully separated the organoids with a crypt‐like proliferative phenotype and the ones with a villus‐like phenotype enriched for enterocytes and goblet cells. By displaying the proteins expressed by the organoids, this study provided a framework for further investigation of the underlying mechanisms of intestinal ischemia–reperfusion injury and promoting the regeneration of specific pathways in crypt‐like organoids.^24^ In a recent study, proteomics discovered several dysregulated proteins from neural progenitor cells from schizophrenic patients‐derived cerebral organoids that can alter and disturb normal neuronal development.^25^ Metabolomics was applied to the kidney organoid derived from human induced pluripotent stem cells (hiPSCs) from healthy patients to investigate the metabolic dynamics and function during kidney organoid differentiation.^26^ It was validated that the dominant metabolic alteration was from glycolysis to oxidative phosphorylation in the hiPSC differentiation process. Additionally, glycine, serine, and threonine metabolism had a regulatory role during hiPSC‐derived kidney organoid formation and lineage maturation. Metabolomics was also applied to human endometrial epithelial organoids to distinguish the donor differences in endometrial epithelial cells with a greater resolution.^27^ Accordingly, we summarized some representative examples of multiomics analysis on hPSC‐derived organoids (Table 1).

**TABLE 1:** Summary of multiomics analysis for hPSC‐derived organoids.

Methods	Purposes	Study objectives	References
Transcriptomics	Profiling transcriptomes Deciphering transcriptional activities and pathways	Explicating the expression of hPSC retinal organoid differentiation markers and mRNA alternative splicing Determining organoid‐to‐organoid variability Quantifying organoid cell resemblance to primary tissue equivalents	[15, 16, 21]
Proteomics	Examining proteins to determine their identity, abundance, and function Investigating their identity, quantity, and role in relation to cells, tissues, or organisms	Differentiating between crypt‐like and villus‐like formations in hPSC‐derived small intestine organoids Discovering dysregulated proteins in hiPSC‐derived Schizophrenic‐sourced cerebral organoids	[24, 25]
Metabolomics	Analyzing metabolites to provide insights into metabolic processes	Investigating metabolic dynamics and function in hiPSC‐derived kidney organoid differentiation Distinguishing the donors for human endometrial epithelial organoids	[26, 27]

### 2.2 Microscopic image analysis

In the context of hPSC‐derived organoids, phase contrast, and fluorescence microscopy are commonly used for both fixed immunostaining and live‐cell imaging. Fluorescence microscopy has contributed significantly to the characterization of the cellular composition of organoids and their phenotypic resemblance to their original tissues, by labeling specific targets with fluorescent dyes to visualize their distribution within the organoid under a fluorescent microscope.^28^, ^29^ Recent advancements in imaging techniques have made it possible to visualize 3D organoid structures using confocal/two‐photon microscopy and tissue clearance techniques at high‐penetration depths without requiring tissue sectioning.^30^ For instance, tissue clearing has been applied to hiPSC‐derived ureteric bud organoids using the Clear, Unobstructed Brain/Body Imaging Cocktails and Computational Analysis (CUBIC),^31^, ^32^ allowing researchers to visualize epithelial polarity and tubular lumen and repeat branching morphogenesis.^33^ Additionally, the passive clearing technique (PACT) has been optimized for 3D imaging of intact hiPSC‐derived retinal organoids, enabling researchers to visualize the fine morphology and structural organization of photoreceptor cells and bipolar cell layers.^34^

Recently, live‐cell imaging of hPSC‐derived organoids has been increasingly utilized to track organoid formation and functional measurement. For instance, phase‐contrast microscopy has been used to track the morphology formation of hPSC‐derived cerebral organoids for over 58 days, revealing neuroepithelial buds with limited areas of disorganized migratory cells.^35^ Similarly, live‐cell imaging has been used to measure the contractility, calcium transient, and action potential of cardiac organoids.^18^, ^36^ Similarly, researchers have utilized calcium imaging to study sophisticated, self‐organized human brain network activity in cerebral organoids, including both synchronized and non‐synchronized patterns.^37^ Fluorescent gene reporters are also encoded in hPSC lines for in situ tracking gene/protein‐specific cell differentiation and localization in hPSC‐derived organoids.^38^ To further investigate the differentiation and development of cardiovascular cells, green fluorescence protein (GFP)‐TNNT2 and mOrange fluorescence protein (mOrange)‐VE‐Cadherin are used along with long‐term live‐cell imaging.^18^ Reporter gene systems have been used to trace the cell ontogeny of a brain organoid based on somatic mutations at a molecular level.^39^

## 3 INTEGRATION OF AI WORKFLOW WITH ORGANOID SYSTEMS

**FIGURE 2:**
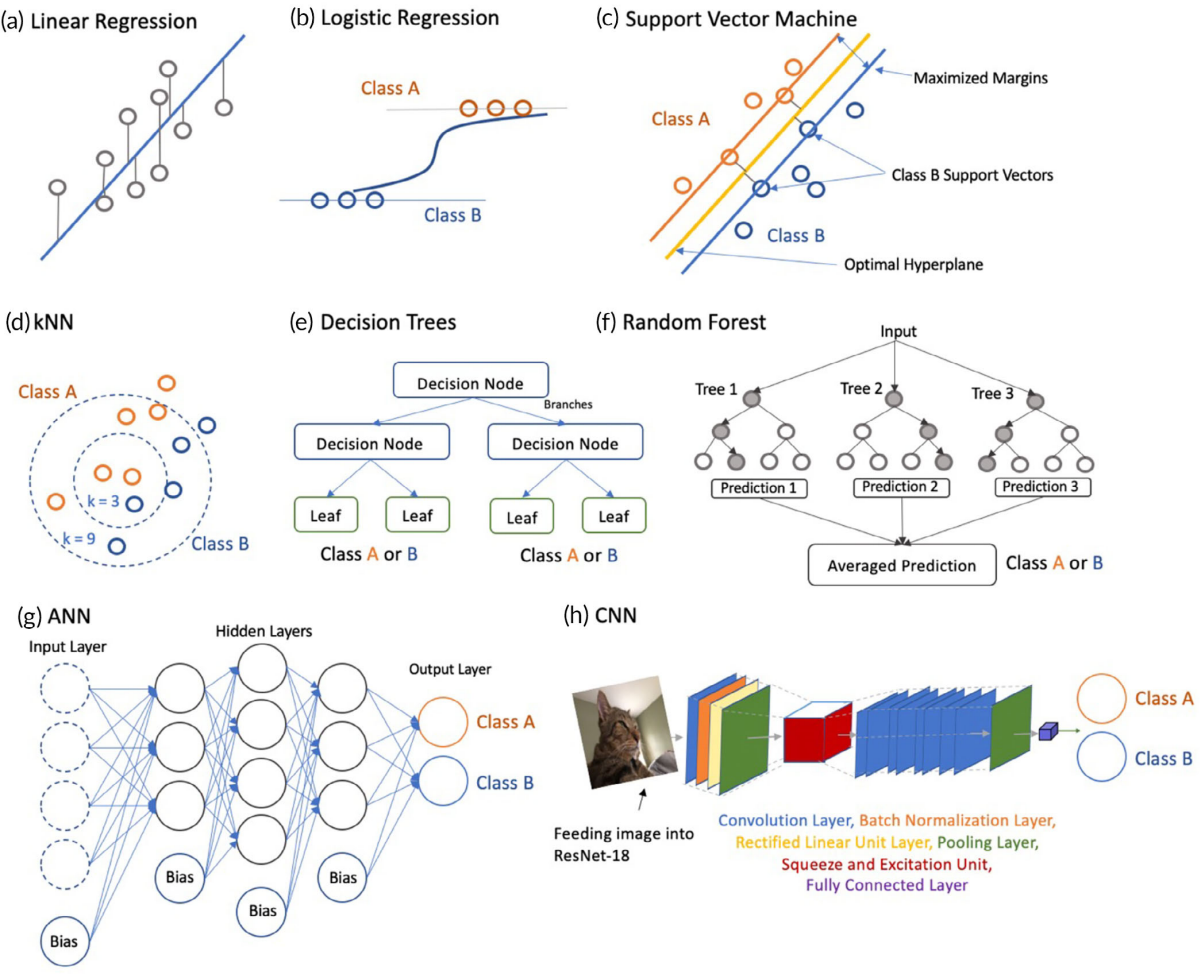
Overview of AI algorithms used in hPSC‐derived organoid research. (a) Linear regression for linear fitting. (b) Logistic regression for binary classification. (c) Support vector machine (SVM) showing maximized margins to determine an optimal hyperplane for classification purposes. (d) kNN with different *k*‐values for classification purposes. (e) Simple 2‐layerdecision tree hierarchy, (f) which can be further expanded into a random forest for classification purposes. (g) Artificial Neural Network (ANN) comprised of 10 neurons in three hidden layers with bias factors for classification purposes. (h) Convoluted Neural Network (CNN) with a 18‐layer ResNet architecture for image classification.

Manual or semi‐manual methods for the hPSC‐derived organoid characterization and analysis are becoming increasingly inefficient as the amount and complexity of the data continue to grow. Advancements in computer and data science have led to the development of numerical automatic methods for interrogating and analyzing organoids using AI algorithms that can observe patterns in datasets and then make predictions.^40^ While the fundamentals of AI are rooted in statistics and complex mathematics, individuals interested in AI applications can take advantage of free, online libraries, such as TensorFlow, Scikit‐Learn, Keras, PyTorch, and Theano, which do not require a deep understanding of AI principles. These accessible AI libraries enable dataset unraveling, pattern identification, and predictive insights in organoid research.^41^ Moreover, they automate labor‐intensive tasks like image analysis, cell tracking, and organoid classification, reducing human errors and optimizing efficiency.^42^ Herein, we provide a general workflow of how to implement AI techniques in organoid studies with three steps as shown in Figure 1, including data preprocessing, dataset construction, model selection of hyperparameter tuning, and data analysis and validation methods. We also provide corresponding illustrations to summarize the ML and DL algorithms that are commonly used in hPSC‐derived organoid research (Figure 2).

### 3.1 Dataset construction and preprocessing

Raw data, such as organoid images and relevant text files, are combined to create a comprehensive dataset for AI models to produce conclusive results. Input data, known as features, represent measurable properties of the samples in a dataset. Selecting the right features is crucial before training a machine learning (ML) model.^43^ While not always necessary, output labels indicate what the model predicts with input data. The dataset is divided into training and testing sets for model instruction and performance evaluation. Features generally fall into two categories: numeric and categorical variables. Most algorithms require data to be presented numerically to be processed effectively. If the data is non‐numeric, categorical labels need to be preprocessed for AI implementation using common techniques, such as one‐hot encoding and ordinal encoding. One‐hot encoding represents categorical variables using binary arrays. For example, in a study predicting cell types within an organoid image, if there are three distinct cell types, each value in a 3‐length array signifies a category (present or not present). For instance, if cell type one is present, the array is [1, 0, 0]. If both cell types one and three are present, the array becomes [1, 0, 1].^44^ In contrast, ordinal encoding is employed when there is a relationship among distinct input types. It assigns integers to each data category instead of binary values, establishing a ranking that the model can utilize for predictions.

### 3.2 Supervised machine learning models

Processed datasets are employed to train and validate AI models, ensuring their suitability for testing environments. The choice of AI models depends on the problem and data structure, primarily utilizing supervised or unsupervised learning depending on the availability of target data for prediction.^45^ Supervised learning is applied when datasets include input and target output pairs. During training, adjustments are made to align the model's predictions with true target values. For example, a trained ML model can categorize input and classify if an image of a brain organoid contains a tumor or not.^40^ Model accuracy, determined by the percentage of correct classifications over total classifications made during training, is a common evaluation metric, though various additional metrics are valuable, especially for multiclass classification problems.

Selecting the appropriate ML model is crucial for the success of organoid analysis. There is a wide array of ML algorithms to consider, each tailored to different problem types. Linear regression, a fundamental supervised learning method, excels in modeling complex relationships and conducting feature selection via regularization techniques like Lasso and Ridge regression. However, it is constrained by its linearity and dependence on well‐defined hypothesis functions. In contrast, logistic regression handles classification tasks by constraining outputs between 0 and 1, making it versatile for multi‐class scenarios.^46^ Naive Bayes, akin to logistic regression, predicts class labels based on joint probability calculations using Bayes Theorem, assuming feature independence, though this assumption may limit its effectiveness in complex or correlated datasets. Support‐Vector Machines (SVM) aim to maximize class separation by identifying optimal hyperplanes, with the capacity to employ non‐linear kernels, but they may overfit and demand significant computation, limiting suitability for large, intricate datasets. k‐Nearest Neighbors (k‐NN) relies on geometric proximity for classification, featuring tunable parameters for neighbor count and distance metric. However, it is computationally intensive, best suited for smaller datasets, and susceptible to overfitting in high dimensions. Decision trees, in contrast to traditional linear classifiers, can capture non‐linear decision boundaries through recursive branching into conjectures. They split based on data labels, with depth determining decision granularity. To enhance accuracy, random forests were introduced, introducing randomness via feature dimension exclusion, and their decisions are averaged for a more generalizable model. However, the recursive nature of tree structures can lead to overfitting, especially with an excessive number of trees.

In an early study that applied ML models to hPSC‐based cardiac research, different ML models were tested to distinguish between normal and abnormal Ca^2+^ signals collected from hiPSC‐derived cardiomyocytes (hiPSC‐CMs). Compared with discriminant analysis, naive Bayes, decision trees, and the k‐NN model achieved up to 80% accuracy in classifying the signals.^47^ A more recent study also evaluated different ML models, including decision trees, quadratic discriminant analysis, SVM, k‐NN, and naive Bayes, to differentiate the contractile profiles of hiPSC‐CMs from either healthy wild‐type controls or a patient with Timothy Syndrome. In this study, decision trees and quadratic discriminant analysis achieved the highest accuracy at 92%, surpassing SVM and k‐NN at 91%.^48^ These studies demonstrate the importance of testing multiple ML models for better performance, due to the differences in classification purposes or size and structure of the original dataset. These approaches can be readily applied to the field of hiPSC‐CMs for enhancing evidence‐based decision‐making in drug development and disease modeling, by analyzing complex datasets in an objective, sensitive, automated, and user‐independent fashion.

### 3.3 Unsupervised learning

Unsupervised learning is instrumental in drawing insights from input data and identifying correlations or patterns that experts can later analyze and leverage for their objectives. Utilizing unlabeled data is generally more cost‐effective and less time‐consuming, as data annotation by experts is unnecessary.^49^ Common unsupervised ML approaches, such as dimensionality reduction and clustering, are commonly used together in a data analytics workflow. Dimensionality reduction techniques reduce the number of dimensions or features in a dataset by embedding the higher dimensional data structures into a lower dimensional space while maintaining the data's structure in the new projected space called the latent manifold. Meanwhile, data clustering helps reveal similarities and differences in the features from different samples. Given that ML algorithms typically require more samples than features for prediction tasks, unsupervised dimensionality reduction can create optimal data representations for subsequent data clustering or supervised learning.^50^

Principle component analysis (PCA), as a tractional dimensionality reduction technique, only focuses on the linear relationship within the data structure and projects the variation into a reduced feature space,^51^ while newer developments, such as multidimensional scaling,^52^ isomaps,^53^ locally linear embedding,^54^ and t‐distributed stochastic neighbor embedding (t‐SNE),^55^ utilize nonlinear transforms to preserve the pairwise distance between points projected into lower dimensional space. Currently, the most used technique, uniform manifold approximation (UMAP), is built upon t‐SNE by introducing repulsive forces between points into their latent manifolds to better preserve global data structure.^56^ After data dimensionality reduction, clustering algorithms are required to identify clusters within the feature space. Hierarchal clustering algorithms are built by recursively splitting pairs of data by similarity to their closest neighbor until all pairs have been split.^57^ k‐means clustering separates available data into a k‐number of clusters by overall similarity based upon relative averages, making it very useful when the number of presumed clusters is known.^58^ Similar to hierarchal clustering, density‐based spatial clustering of applications with noise (DBSCAN), or its adaptive application (ADBSCAN), does not require a researcher to specify the number of clusters. DBSCAN can be adapted to datasets that do not have clear clusters by locally focusing on the relative density of points and automatically ignoring the outliers within the feature space.^59^

Both dimensionality reduction and data clustering have been extensively utilized in single‐cell transcriptomics analysis. Recently, these data‐driven analytical techniques have been explored for analyzing the phenotypic properties of hiPSC‐CMs. For example, a non‐linear dimensionality reduction technique, uniform manifold approximation and projection (UMAP), was employed to project the contractility waveforms generated from beating hiPSC‐CMs into a two‐dimensional (2D) space for visualizing and clustering the cells with different contractile behaviors. In addition, fast Fourier transform (FFT)‐based data preprocessing could enhance the performance of an SVM model in classifying a contractility waveform between normal and abnormal ones.^60^ In a hiPSC‐CM cardiotoxicity study, three ML models, SVM, random forest, and neural network, were used to classify three different drugs (verapamil, isoproterenol, or cisapride). Results showed that the neural network outperformed the other two models with an initial accuracy of 71.4% in drug classification, which was boosted to 80% accuracy with the addition of data preprocessing steps. In addition, t‐SNE, a dimensionality reduction technique, was used to visualize how data preprocessing can help the separation of drug effects and allow ML algorithms to detect subtle variations among different drugs.^61^ In another study, the t‐SNE algorithm was used to investigate the structure–function relationships of cardiac organoids generated from different micropattern sizes. This data visualization technique allowed us to identify the correlation between pattern size and parametric functional parameters of cardiac organoids, revealing important associations.^62^

### 3.4 Deep learning neural networks

Artificial neural networks (ANNs) have evolved since the late 1950s into sophisticated frameworks today.^63^, ^64^ ANNs consist of interconnected layers of neurons that process inputs, like images, through hidden layers with randomized weights before reaching the output layer. Neurons compute outputs using activation functions, and weights control information flow. Backpropagation with gradient descent aids weight updates, though challenges like vanishing and exploding gradients can occur. Deep learning (DL), a subset of artificial neural networks (ANNs), employs layered neural networks that process data in sequential stages, akin to the human brain, transforming input from low‐level to high‐level features for predictive tasks.^65^ Although DL demands substantial computational power, large datasets, and lengthier training periods compared with standard ML models, it automatically extracts features, eliminating the need for manual feature identification—particularly advantageous for processing unstructured data like images and audio, where manual labeling can be impractical and yield inaccurate results.^66^

Particularly, convolutional neural networks (CNNs) have gained much attention in the fields of hPSCs and organoids for their capability of extracting image features through convolutional layers, ensuring accurate classification. For example, a CNN‐based image analysis system integrated classification, segmentation, and statistical modeling to measure morphological dynamics during hiPSC reprogramming and guide colony selection in a label‐free non‐invasive manner. The time‐lapse bright‐field images were processed using a sliding window, and each window image was then classified by a CNNs model to detect the earliest cellular texture changes after the induction of reprogramming in human somatic cells. Verified by an OCT4‐GFP reporter cell line, this trained CNNs model was able to predict distinct phases of colony formation during hiPSC reprogramming and identify the optimal phase for colony selection, as a practical solution for analyzing large datasets where fluorescence reporting is inefficient and susceptible to human error.^67^, ^68^, ^69^, ^70^ In a study of brain organoids, a deep CNN was trained to classify immunofluorescent images of wild‐type (WT) and Huntington's Disease (HD) neuruloids generated using micropatterning techniques.^71^ The CNN's image classification prowess enabled precise phenotypic categorization despite biological noise, allowing near‐perfect discrimination between WT and HD at the individual neuruloid level and per‐well average score. Statistical assessment, including Z0 factor comparison with other ML methods, affirmed model effectiveness in discrimination.

### 3.5 Hyperparameter tuning and model validation

Hyperparameters wield significant influence over the accuracy and efficiency of ML outcomes.^72^ In some cases, tinkering with hyperparameters can yield effects comparable to redesigning the entire ML model.^73^ For instance, when fitting the data points using regression approaches, the degree of polynomials (a linear, quadratic, or cubic function) is a hyperparameter to be considered. Hyperparameters serve as a blueprint for the model's architecture, dictating crucial aspects such as the number of branches in a decision tree, clusters in a clustering algorithm, or the number of neurons and layers in a deep neural network. Unlike model parameters, which evolve autonomously throughout the training process to fit the input–output relationships, hyperparameters are typically set manually or optimized via validation processes before training commences.^74^, ^75^

Hyperparameters directly influence model complexity, which is critical since some datasets are prone to overfitting or underfitting. Optimizing hyperparameters aims to attain the model's best performance. This can be accomplished manually through trial and error, while automated methods like grid search and random search provide systematic ways to discover optimal hyperparameter combinations for ML models.^76^ For instance, in a methodology combining computational analysis and cardiac organoids to replicate heart development in both healthy and pathological conditions, hyperparameters played a critical role in classification functions for cell type, anatomical zone, and laterality.^77^ In essence, hyperparameter optimization becomes an indispensable step in harnessing the full potential of AI models, allowing researchers to tailor their ML algorithms to intricately match the complexity and nuances of their datasets and research objectives.

Validation methods play a pivotal role in the training process, as they assess ML model performance on unseen data through various metrics (accuracy, precision, and mean root square error), indicating the model's ability to generalize with new data.^78^ Among these metrics, the error rate stands out as a critical indicator for model predictivity. Commonly used in supervised learning, two prevalent validation techniques are *k*‐fold cross‐validation and leave‐one‐out cross‐validation (LOOCV). In *k*‐fold cross‐validation, the dataset is divided into *k* groups, with one group serving as the testing set and the other *k* − 1 groups as the training set. This process is repeated *k* times, with each group serving as the testing set once, and the results are averaged to evaluate model adaptability to new data.^79^ LOOCV, a variant of *k*‐fold cross‐validation, assigns each data point in the dataset of size *k* as the testing set, while the rest of the data is the training set. This process repeats *k* times, so every single data point serves as the testing set once, allowing for a comprehensive assessment of the model's performance.^80^ However, LOOCV becomes impractical with large datasets, owing to its high computational demands and time‐consuming nature.^81^

## 4 AI‐ENABLED ANALYSIS FOR hPSC‐DERIVED ORGANOIDS

The abundance of multidimensional data from high‐content and high‐resolution imaging, multiomics, and functional assays presents challenges in correlation and analysis. AI has emerged as a promising solution, meeting the demands, and assisting in overcoming these challenges in the field of hPSC‐derived organoids. With advancements in computer processing capacities and more sophisticated algorithms, ML/DL can provide more efficient and nuanced analytical approaches,^82^, ^83^, ^84^ which can help unravel the complex interplay of biological factors and gain mechanistic insights in organoid research.^63^ Here, AI applications in hPSC‐derived organoid models for various biomedical applications are summarized in Table 2.

**TABLE 2:** AI‐enabled data analysis for hPSC‐derived organoids.

Data type	Organoid type	Study objectives	References
Omics data	hiPSC‐derived cardiac organoids hiPSC‐derived brain organoids hiPSC‐derived hypothalamic organoids hiPSC‐derived neural organoids	RF for analyzing single‐cell RNA‐seq datasets of cell (sub)types in the heart kNN for comparatively analyzing single‐cell RNA‐seq datasets from brain tissue and hiPSC‐derived organoids Seurat transfer learning workflow for identifying activity‐regulated cytoskeleton‐associated proteins in hiPSC‐derived hypothalamic organoids SVM for identifying neurotoxicity of chemicals within the hiPSC‐derived neural organoids	[77, 85, 86, 87]
Time‐series function data	hiPSC‐cardiomyocytes	SVM, kNN, and RF classifiers for automatic cardiac function assessment of Ca^2+^ transient abnormality CNN for detecting and quantifying arrhythmic waveforms of cardiac action potential	[88, 89, 90, 91, 92]
Image data	Intestinal organoids hiPSC‐derived brain organoids hiPSC‐derived retinal organoids hiPSC‐derived kidney organoids	CNN for automated detection of cell nuclei from single microscopy image slices CNN for automated quantification size and localization of organoids from bright‐field images RF for automated classification of control and 6‐OHDA treated organoids from fluorescence image of neuronal network Logistic regression or multi‐layer perceptron models for morphology quantification of bright field and fluorescence images of organoids CNN for organoid subcategory classification from bright‐field and fluorescence images CNN for predicting the differentiation of kidney organoids on bright‐field images	[44, 93, 94, 95, 96, 97, 98, 99]

Abbreviations: 6‐OHDA, 6‐hydroxydopamine; CNN, convolutional neural network; kNN, k‐nearest neighbors algorithm; RF, random forest.

### 4.1 Enhancing comparative omics analysis

AI serves as a potent tool that greatly enhances the analysis, interpretation, and practical utilization of multidimensional omics data within the realm of biomedical science and engineering. One innovative application involves the utilization of synthetic data generated through generative ML models.^100^, ^101^, ^102^ This synthetic data can be instrumental in benchmarking various stages of the analytical pipeline, including sample processing, multidimensional separation, and data acquisition, regardless of whether the sample has been previously processed. By doing so, it effectively replaces guesswork in determining optimal acquisition parameters, particularly when dealing with single‐cell analysis or other valuable biological and clinical specimens. Taking the field of proteomics as an example,^103^ platforms like ProteomicsML.org have emerged as a valuable online proteomics data repository with companion tutorials designed to facilitate the training of ML models. It is noteworthy that similar resources are continually evolving in various omics domains and building synergy with the ML community, thus fostering an environment where ML experts can readily experiment with omics data, while omics specialists can explore and harness the capabilities of ML applications. Although there are still a limited number of reported studies on AI‐based analysis for multiomics datasets collected from hPSC‐derived organoids, this collaborative effort can be easily expanded to drive forward our understanding of complex biological systems and expedite breakthroughs in the technological integration of omics, AI, and hPSC‐derived organoids.

ML approaches have been used to evaluate the transcriptomic proximity of organoids for modeling human organ development. To investigate heart development in both healthy and diseased conditions, a workflow was established by combining heart organoids, single‐cell RNA sequencing, and ML. Atrial‐lineage and ventricular‐lineage heart organoids were generated with and without retinoic acid respectively. By employing single‐cell RNA sequencing and sample multiplexing, different cardiac cell types were characterized in these chamber‐specific organoids. Meanwhile, an ML model based on random forest (RF) was implemented to leverage the information about cell types, heart chamber (atria vs. ventricle), and laterality (left vs. right) available in the cell atlas of primary human fetal hearts and transfer cell annotation into this organoid system. This method can perform anomaly detection to filter out cell types that were not present in the training data, allowing to focus on cardiac cells from the organoids in the comparison between a genetic variation in NKX2‐5 associated with Ebstein's abnormality and its isogenic control.^77^

Various studies have highlighted the potential of using organoids to model brain development, yet the fidelity of these models has sparked debate due to a lack of computational tools for comprehensive gene expression analysis across developmental stages in both human brains and organoids, particularly for single‐cell datasets. In response, A manifold‐learning framework, Brain and Organoid Manifold Alignment (BOMA),^85^ was designed to bridge this gap by aligning transcriptomic data between brains and organoids. Using a kNN‐based ML technique, BOMA maps the manifolds cross multiple datasets from primary brain tissues and brain organoids and projects them into a common latent space to uncover developmental trajectories either conserved from aligned data or distinctive from unaligned data. Using this method, different brain organoids were found closer to certain brain regions at specific time points. In addition, by aligning the scRNA‐seq data from human and chimpanzee organoids, a delayed development of human organoids was determined in comparison to chimpanzee organoids. Although this study only focused on RNA‐seq datasets between brains and organoids, this framework can be easily applied to compare any sample pairs, such as different modalities or different organ‐organoid systems.

### 4.2 Feature engineering from time‐series data

In various real‐world applications, data collected over time often forms time‐series datasets with inherent temporal dependencies, adding complexity to their analysis. Historically, addressing this challenge relied on crafting features manually, which was resource‐intensive and required domain expertise. For example, an electrocardiogram (EKG) is one of the classic time‐series data, offering valuable insights into the heart's electrical activity over time. This time‐dependent nature of EKG data lends itself to advanced ML and data analysis techniques, allowing for the detection of anomalies, arrhythmia classification, event prediction, and overall cardiac health assessment making it indispensable in healthcare and clinical applications. Similarly, in the field of cardiac organoids, functional outputs of beating hiPSC‐CMs can be quantified as time‐series datasets to represent their physiological behaviors.^88^, ^90^, ^91^ The measurement of contractile motions, action potentials, and intracellular calcium transient produces time‐series datasets, which can be analyzed using traditional peak detection methods, while more recently, ML approaches have been applied for better assessment of cardiac physiology for these hiPSC‐based in vitro models.

For instance, a new analytical pipeline for automatic assessment of Ca^2+^ transient abnormality was developed by employing ML methods together with an analytical algorithm. Ca^2+^ transient data from 200 hiPSC‐CMs with 1893 peaks was used to train a peak‐based SVM model, which was then integrated with 15 peak‐related features extracted from an analytical algorithm to further develop a cell‐based SVM model. This trained cell‐based SVM classifier was tested on an additional 54 cells and obtained a testing accuracy of 87% for detecting abnormality. This algorithm has the potential to automate the Ca^2+^ transient analysis and assist the decision‐making of signal abnormality, regardless of its origin and experimental procedures employed.^104^ Based on the abnormality of Ca^2+^ transient signals, it is possible to distinguish the healthy and diseased hiPSC‐CMs with different genetic deficiencies. In a study with six iPSC lines carrying different mutations causing catecholaminergic polymorphic ventricular tachycardia (CPVT), hiPSC‐CMs were treated with adrenaline and dantrolene to test anti‐arrhythmic effects.^105^ Twelve peak features from Ca^2+^ transient signals were computed, z‐normalized, and input for SVM, k‐Nearest Neighbors Algorithm (kNN), and RF classifiers. The RF classifier achieved an overall accuracy of 65.6% in classifying drug responses into responding, semi‐responding, and non‐responding. In the following study with 1635 peaks of long QT syndrome (LQT1), 1344 peaks of hypertrophic cardiomyopathy (HCM), 2311 peaks of CPVT, and 1216 peaks of healthy controls (WT), different ML models, including k‐NN, RF, and SVM, were used to classify the Ca^2+^ transient signals in respect to the disease types. The overall accuracy achieved by these algorithms in distinguishing different diseased lines was over 80%, with the random forest classifier performing the best at 87.6% accuracy.^89^ These proof‐of‐concept studies demonstrated that analyzing time‐series data from Ca^2+^ transient using ML techniques can accurately categorize hiPSC‐CMs based on their drug responses and genotypes for future applications in drug screening, disease modeling, diagnostic practice, and personalized medicine.

Previous approaches to utilizing time‐series cardiac physiological data for ML involved feature extraction, which demanded expertise in signal processing techniques and risked losing subtle but valuable information. Alternatively, unsupervised ML has emerged as a powerful method to automatically learn feature representations from unlabeled data, eliminating the need for labor‐intensive hand‐crafted data processing. These learned feature representations can be stacked to construct deep neural networks capable of modeling intricate data structures. In a recent study, a CNN model was trained using membrane potential data from hiPSC‐CMs to extract 65 machine‐learned features, facilitating the classification of voltage traces into non‐arrhythmic, arrhythmic, and asystolic categories.^92^ This approach enabled the plotting of dose‐dependent proarrhythmic curves for each drug, yielding EC50 values. Subsequently, the torsadogenic safety margin for each drug was calculated based on the ratio between the EC50 value and *C*_max_, the maximum free plasma concentration. This safety margin served as a predictor in a logistic regression model to distinguish high‐, intermediate‐, and low‐risk compounds. The CNN‐based platform effectively captured time‐series voltage trace features from hiPSC‐CMs, enabling the quantification of the transition from normal to arrhythmic waveforms in response to drug dose—a capability unattainable through traditional binary arrhythmia assessments relying on human judgment. Moreover, the calculated safety margins demonstrated enhanced accuracy in discriminating high‐, intermediate‐, and low‐risk drugs compared with previous methods relying on human‐defined features. While most of the studies are conducted in the hPSC‐derived cells, the similar AI technologies could be potentially applied in the hPSC‐derived organoids, which are able to be characterized with time‐series function measurements.

### 4.3 Image analysis using deep learning techniques

The analysis of image data from hPSC‐derived organoids presents significant challenges, particularly in the areas of cell segmentation and phenotypic annotation. Even experienced image analysis professionals struggle with accurately and efficiently segmenting cells and annotating phenotypes, especially when dealing with densely packed and optically opaque cell aggregates that exhibit strong interactions. ML techniques offer a solution for image‐based profiling and analysis, particularly when combined with advancements in automated microscopy^106^, ^107^ and high‐throughput screening (HTS) platforms.^108^, ^109^ For example, CNN models have been applied in the automated tracking of cell nuclei in intestine organoids from single microscopy image slices of fluorescence nuclei staining.^94^ This method provides a much faster speed of image analysis at the equivalent tracking quality to manual tracking. CNNs are also used in automated quantification size and localization of a large number of hPSC‐derived intestine organoids based on bright‐field images.^93^ In the hPSC‐derived brain organoids, logistic regression or multilayer perceptron models were able to achieve morphology quantification from bright‐field images and reporter gene expression quantification from fluorescence images of hundreds of organoids.^44^

Moreover, high‐throughput and high‐content imaging can meet the requirement of large‐scale data volume required by the DL algorithms to identify the subtle patterns and correlations that may be missed by human observers. AI has been used to optimize compound screening by facilitating predictive modeling for therapeutic reactions.^110^, ^111^ For instance, DL was utilized to directly analyze the 3D image stacks of hiPSC‐derived mammary gland organoids without converting them into 2D projections or specifying individual cell types.^112^ DL‐Based Senescence Scoring by Morphology (Deep‐SeSMo) is a CNN‐based model that uses phase‐contrast microscopy images without molecular labels to generate senescence probability on iPSCs in large numbers.^113^, ^114^

DL methods have been utilized in studies focused on cardiotoxicity to quantify drug‐induced structural changes in hiPSC‐CMs. DL models trained with both brightfield and fluorescent images of hiPSC‐CMs have demonstrated their ability to detect cellular changes resulting in the loss of cardiac function. The early success of neural network models in identifying toxic effects has shown great promise in high‐throughput toxicity screening.^115^ Additionally, convolutional neural networks trained with dose‐dependent images have been effective in detecting changes preceding the loss of contractility in hiPSC‐CMs, indicating the potential of image‐based DL methods in predicting cardiotoxic effects.^116^, ^117^ In a recent study, a high‐throughput screening platform was used to evaluate a library of 1280 bioactive compounds with potential cardiotoxic liabilities, and a DL model was constructed. This model exhibited exceptional capability in identifying chemicals with cardiotoxic effects and effectively classifying the compounds based on distinct mechanisms of action.^116^

A brain organoid is a deliberately developed micro‐organ in vitro that aims to replicate the structure and characteristics of the brain.^118^ Brain organoids are artificial tissues that mimic different cortical areas and consist of various types of nerve cells. The cortex and choroid plexus, two layers of neurons, closely resemble cerebral organoids, while other regions such as the retina, meninges, and hippocampus can also develop to some extent.^119^ Researchers have identified optimized techniques and identified the essential parameters necessary to promote the formation of well‐developed organoids. Their optimization criteria include overall growth and size of organoids, stratification and representation of cell types, inter‐batch variability, analysis of neural maturation, and cost‐effectiveness of the process. These experiments and findings provide a reliable approach for genetic or pharmacological testing (e.g., drug development), which can aid in the better understanding and treatment of human neurodevelopmental disorders and lead to the creation of organoids with reduced variability.^120^ Advancements in electrophysiological recording techniques in vivo, such as Neuropixels,^121^, ^122^ as well as neuroimaging techniques,^123^ have paved the way for analyzing highly specific populations of neurons and brain regions with high spatiotemporal resolution. AI has been widely employed to further enhance hPSC‐derived brain organoids in various applications, taking advantage of these characterization techniques and advancements. The evaluation of neurotoxicity has been significantly improved through the combination of hPSC‐derived midbrain organoids and ML techniques.^96^ The dense clustering of cells and neurons within the organoids made it extremely challenging to manually extract neuronal features through microscopy‐based phenotyping. To address this issue, an ML model was developed using a random forest classifier to automatically discern the differences between control and 6‐hydroxydopamine (6‐OHDA) treated organoids. To minimize bias in the model's predictions, a 10‐fold cross‐validation was applied five times. After normalization, the model achieved an impressive accuracy of 86% in classifying the organoids with or without neurotoxin.

Furthermore, a DL algorithm utilizing CNNs was employed for a classification task involving brain organoids with different morphology types. The aim was to investigate whether erythromyeloid progenitors (EMPs) would migrate to brain organoids in the presence of hiPSC‐derived microglia.^124^ To develop the CNN model, the researchers utilized an AI platform called Aiforia to annotate and distinguish between different morphology types based on immunological staining. AI was able to quantify the number of ramified, intermediate, rod‐shaped, and spheric cells in organoid sections on days 35, 66, and 120. The researchers performed a manual approach using skeletal analysis to measure the complexity of cell morphology, and the results were comparable to those obtained through the AI method. This validation confirmed the reliability of the ML model and highlighted the efficiency of an AI approach in assessing the complexity of structures within organoids. Furthermore, the detection algorithm successfully identified a subset of cells that increased from day 66 to 120, despite the overall decrease in complexity, demonstrating the ability of AI to detect subtle changes within a dataset. By analyzing microglial and neuronal diversity patterns through CNNs, researchers can gain a deeper understanding of the cellular structure and development within brain organoids, providing valuable insights into their complexity and maturation processes.

ML techniques have shown great potential in enhancing the capabilities of brain organoids to predict input factors, optimize data collection and analysis, and decode the functional relationships between input and output. Through disease stratification, ML‐based integration of multimodal data was utilized to improve Parkinson's disease (PD) modeling based on hPSC‐derived brain organoids.^125^ This could potentially comprise in vitro data produced by the organoids from PD patients, which can be integrated with in vivo data of demographics, magnetic resonance imaging (MRI), genetics, and other clinical information.^126^ Notably, the integration of Brain–Computer Interface (BCI) feedback with brain organoid modeling can enable dynamic closed‐loop control by combining ML algorithms and organoid technology.^127^

Multiple research studies have demonstrated that retinal organoids derived from hPSCs closely resemble the histology, cellular specificity, sub‐specification, functionality, and transcription profiling of the human retina.^128^ This highlights the robustness of this technology and its potential for clinical applications, such as providing a significant source of retinal neurons for transplantation^129^ or serving as a platform for testing novel treatments.^130^ To create a comprehensive single‐cell‐resolution map of the human Retinal Pigment Epithelium (RPE), researchers conducted a study using 17 RPE flatmounts obtained from the eyes of nine donors without any notable eye conditions at the time of analysis. The edges of RPE cells were stained with Phalloidin‐iFluor 647. Each RPE flatmount, with an approximate radius of 23 mm, was scanned in 3000 tiled panels at ×20 magnification. The researchers employed REShAPE, an ML‐based program based on a U‐net CNN, which analyzes fluorescence images to detect and segment RPE cell boundaries and performs RPE cell morphometry. The program successfully distinguished and segmented cell boundaries in the images. On average, each flatmount contained around 2.8 million RPE cells, all of which were identified and individually segmented. Using the resulting binary image of RPE cell boundaries, morphometric characteristics were calculated for every single cell in the entire human eye.^131^ This groundbreaking research has enabled the generation of a detailed understanding of the cellular characteristics and organization of the human RPE at the single‐cell level. It showcases the potential of ML‐based approaches, such as REShAPE, in analyzing large‐scale imaging datasets to extract valuable insights and contribute to the advancement of retinal research and clinical applications.

DL‐based automated differentiation of retinal organoids was successfully achieved using CNNs such as VGG19, ResNet50v2, DenseNet121, and Xception.^132^ The CNNs were trained to classify bright‐field and fluorescence images, including those with a GFP reporter, into three categories: retina, satisfactory, and non‐retina. Each data sample was assigned a probability rating between 0 and 1, indicating the likelihood of it being a retina image. The CNNs achieved an average accuracy rate of 0.84, surpassing human classifiers who achieved an accuracy of 0.67. This highlights the superior performance of DL in classification tasks, enabling the efficient processing of large amounts of data and accurate prediction of the early stages of organoid development.

Furthermore, by employing image recognition and ML techniques, heterogeneous retinal organoids can be “normalized” and categorized based on their sizes, hues, fluorescent protein‐tagged markers, or other properties ^98^. This normalization process reduces variations during drug testing, disease modeling, developmental research, and other procedures, thereby enhancing the consistency and reliability of organoid studies. Furthermore, DL techniques were utilized to generate high‐quality images of hPSC‐derived retinal organoids while minimizing the number of scans and reducing phototoxicity caused by two‐photon excitation fluorescence.^95^ The primary approach employed path‐based regression, which involved dividing each input image into smaller tiles and training a neural network on these tiles. The results showed a mean structural similarity index measure (SSIM) value of 0.64, indicating the potential of this approach for future applications. The combination of image recognition and ML algorithms provides a valuable tool for streamlining and standardizing the analysis of retinal organoids, leading to improved efficiency and reproducibility in research and potential applications.

## 5 CONCLUSIONS AND FUTURE PERSPECTIVES

**FIGURE 3:**
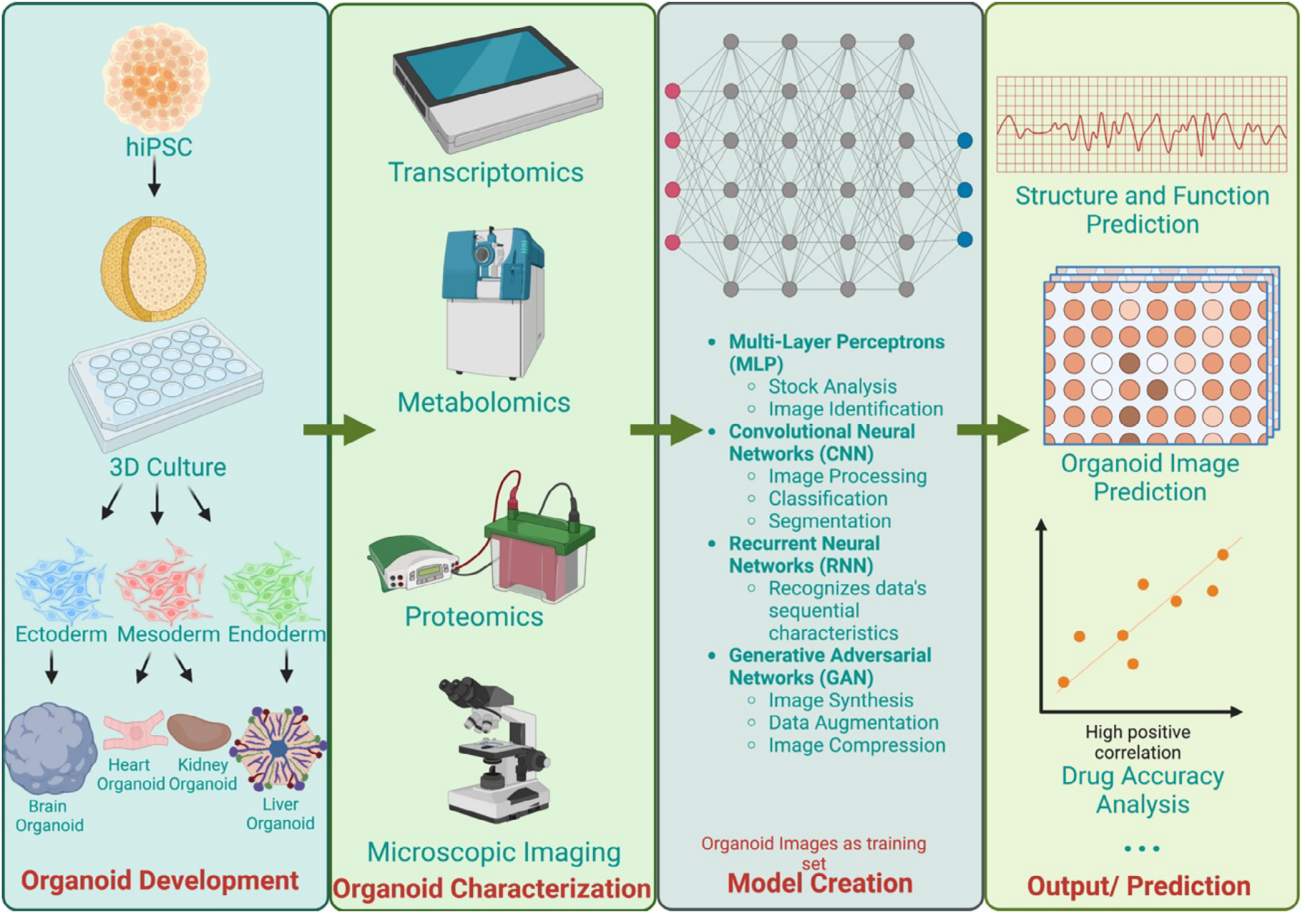
AI applications in hPSC‐organoids. AI‐enabled hPSC organoid research comprises a sequential workflow of organoid development and characterization, AI model establishment and optimization, and AI‐driven data analytics and predictions on organoid structural morphology, functional outputs, and drug responses.

The development of hPSC‐derived organoids presents both opportunities and challenges in modeling tissue/organ development and disease. Increasing sample numbers and complexity in the traditional approaches, even with automation and scale‐up techniques, may not provide a deeper mechanistic understanding. AI has emerged as a valuable tool in evaluating organoids in various areas, such as disease modeling,^133^ drug evaluation,^92^, ^105^ and regenerative medicine,^134^ due to its ability to extract meaningful insights from organoid traits and process large volumes of data efficiently. For example, the studies of hPSC‐derived organoid maturation would be assisted by AI technologies to generate the optimal organoid maturation protocol based on multiple physiological features of corresponding organs or tissues, for feature importance analysis, supervised classification, and unsupervised clustering. Currently, quality control of organoid development relies on end‐point post‐differentiation measurements. If ML could predict the expected results from different samples during the early stages of organoid differentiation, it could guide experimental planning and execution, thus greatly improving the quality and reliability of organoid sources. Moreover, generating an experimentally relevant synthetic ground‐truth dataset of organoid differentiation and functionality will allow for benchmarking and identifying best‐performing differentiation approaches and culturing conditions.^97^, ^132^, ^135^ Recently, we applied the function of feature importance to rank the features to determine the most effective growth factors and small molecules for cardiac differentiation and vascularization in the hPSC‐derived cardiac organoids.^136^ In this review, we discuss an AI framework tailored to biomedical research, particularly focusing on hPSC‐derived organoids (Figure 3), aiming to enhance our comprehension of hPSC‐derived organoids with improved efficiency and precision.

AI‐based methods enable unbiased measurement of a wide range of cellular characteristics and capture subtle variations under different conditions.^137^, ^138^ Cell painting and other phenotype‐agnostic staining processes enhance label‐free cell‐type recognition and enable a non‐invasive analysis of cell populations, which differs from traditional image‐based methods only focusing on predetermined phenotypes.^107^, ^139^, ^140^, ^141^ These “in silico labeling” AI techniques were used to predict and identify multiple fluorescent markers, such as live/dead labeling and antibody staining, from transmitted‐light microscopy data, ensuring flexibility and efficiency in image analysis. For example, DL models were created to recognize unstained hPSC‐derived endothelial cells in phase‐contrast images, solely based on their distinctive shape.^142^ Moreover, these technological breakthroughs in AI can surpass human perception and analytical capabilities in clinical research. For instance, AI has demonstrated superior performance to clinical experts in the interpretation of medical images for detecting retinal disorders,^143^ skin conditions,^144^ lung abnormalities,^145^ and breast malignancies.^146^ These advancements have significant implications for identifying heterogeneous cell populations within hPSC‐derived organoids without fluorescent labeling processes. It can help determine the differentiation efficiency at the early stage of organoid formation or identify cell deficiency in organoids under diseased conditions. By leveraging ML and image‐based profiling techniques, researchers can overcome the challenges associated with complex image data collected from organoids, enabling comprehensive and unbiased characterization of organoid features at the cellular level.

ML/DL‐based techniques for image analysis of 3D imaging data are rapidly advancing and becoming more accessible,^147^ which could be critical for further advancement of organoid imaging analysis due to their 3D nature. Flexible analytical tools like NiftyNet have taken advantage of the modularity of modern DL platforms to be applicable to a wide range of imaging modalities.^148^ Another example, CDeep3M, is a cloud‐based ML application capable of processing 2D and 3D data obtained from electron microscopy, x‐ray microscopy, and light microscopy. CDeep3M can fully segment the neuronal processes and synapses in 3D and accurately identify neurons in brain slices based solely on their chromatin structure, even without nuclear staining.^149^ More advanced techniques are being developed for medical imaging analysis. Image reconstruction for MRI has been a long‐studied topic with entire datasets dedicated to furthering DL applications within the field.^150^, ^151^ Computed tomography (CT) has also seen an influx of resolution enhancement,^152^ denoising,^153^ and image reconstruction to fill missing structure data.^154^ For 3D reconstruction in microscopy images, Deep‐Z was developed to extend the depth of view from a single focal layer through a deeper field of view.^155^ Additionally, DL could enhance 2D images to full 3D synthetic images through the use of conditional GANs.^156^ Despite these advancements, there are still very few applications of AI‐based 3D image analysis to organoid models for resolution enhancement, denoising, sub‐structure identification, or image reconstruction with synthetically generated 3D images.

While the future of using AI in organoid research holds promise, several limitations can impede progress. One important consideration is the reliance of AI algorithms on the quality, reproducibility, and integrity of the dataset that they are trained on. High experimental variations and inconsistency in organoid culture and differentiation can pose challenges for AI algorithms in reaching reliable conclusions. For example, the noise present in the microscopy image data could introduce errors and impact the performance of AI algorithms. Moreover, complex biological data obtained from organoids often have significant interrelated factors, but some of them may not have any bearing on the specific task. These AI models have the potential to inadvertently learn and amplify biases present in the data they are trained on. This can result in misleadingly high accuracy rates, as the model may pick up on irrelevant information or subtle correlations that do not truly contribute to the target application's success. To overcome these limitations, it becomes essential to delve into the trustworthy AI model's decision‐making process and thoroughly analyze its outcomes based on the features it relies on. More importantly, it is crucial to continue building a strong experimental foundation and establish a more standardized practice in organoid research to produce well‐organized and reliable data, which allows AI to effectively classify and predict the outcomes from organoid development, drug responses, and disease modeling.

## AUTHOR CONTRIBUTIONS

**Sudhiksha Maramraju:** Visualization (equal); writing – original draft (lead); writing – review and editing (equal). **Andrew Kowalczewski:** Visualization (equal); writing – original draft (lead); writing – review and editing (equal). **Anirudh Kaza:** Writing – original draft (equal). **Xiyuan Liu:** Writing – review and editing (equal). **Jathin Pranav Singaraju:** Writing – original draft (equal). **Mark V. Albert:** Writing – review and editing (equal). **Zhen Ma:** Conceptualization (lead); funding acquisition (lead); project administration (lead); resources (lead); supervision (lead); visualization (equal); writing – original draft (equal); writing – review and editing (lead). **Huaxiao Yang:** Conceptualization (lead); funding acquisition (lead); project administration (lead); resources (lead); supervision (lead); visualization (equal); writing – original draft (lead); writing – review and editing (lead).

## FUNDING INFORMATION

This work was supported by the NIH [R01HD101130 (Z.M.), R15HD108720 (H.X.Y.)], NSF [CMMI‐2130192 and CBET‐1943798], lab start‐up from the University of North Texas (UNT) Biomedical Engineering, Research Seed Grants (2021 and 2023) from UNT Research (H.X.Y.) and Innovation Office, and Syracuse University intramural CUSE grant [II‐3245‐2022 (Z.M.)].

## CONFLICT OF INTEREST STATEMENT

The authors declare no competing financial interest.

### PEER REVIEW

The peer review history for this article is available at https://www.webofscience.com/api/gateway/wos/peer-review/10.1002/btm2.10641.

## Data Availability

Data sharing does not apply to this article as no new data was created or analyzed in this study.
